# Cold Atmospheric Plasma Induces ATP-Dependent Endocytosis of Nanoparticles and Synergistic U373MG Cancer Cell Death

**DOI:** 10.1038/s41598-018-23262-0

**Published:** 2018-03-28

**Authors:** Zhonglei He, Kangze Liu, Eline Manaloto, Alan Casey, George P. Cribaro, Hugh J. Byrne, Furong Tian, Carlos Barcia, Gillian E. Conway, Patrick J. Cullen, James F. Curtin

**Affiliations:** 1 BioPlasma Research Group, School of Food Science and Environmental Health, Dublin Institute of Technology, Dublin, Ireland; 2 Nanolab, FOCAS Research Institute, Dublin Institute of Technology, Dublin, Ireland; 3 Environmental, Sustainability and Health Research Institute, Dublin Institute of Technology, Dublin, Ireland; 4 Institut de Neurociències & Department of Biochemistry and Molecular Biology, School of Medicine, Universitat Autònoma de Barcelona, Barcelona, Spain; 5 Department of Chemical and Environmental Engineering, University of Nottingham, Nottingham, UK

## Abstract

Gold nanoparticles (AuNP) have potential as both diagnostic and therapeutic vehicles. However, selective targeting and uptake in cancer cells remains challenging. Cold atmospheric plasma (CAP) can be combined with AuNP to achieve synergistic anti-cancer cytotoxicity. To explore synergistic mechanisms, we demonstrate both rate of AuNP uptake and total amount accumulated in U373MG Glioblastoma multiforme (GBM) cells are significantly increased when exposed to 75 kV CAP generated by dielectric barrier discharge. No significant changes in the physical parameters of AuNP were caused by CAP but active transport mechanisms were stimulated in cells. Unlike many other biological effects of CAP, long-lived reactive species were not involved, and plasma-activated liquids did not replicate the effect. Chemical effects induced by direct and indirect exposure to CAP appears the dominant mediator of enhanced uptake. Transient physical alterations of membrane integrity played a minor role. 3D-reconstruction of deconvoluted confocal images confirmed AuNP accumulation in lysosomes and other acidic vesicles, which will be useful for future drug delivery and diagnostic strategies. Toxicity of AuNP significantly increased by 25-fold when combined with CAP. Our data indicate that direct exposure to CAP activates AuNP-dependent cytotoxicity by increasing AuNP endocytosis and trafficking to lysosomes in U373MG cells.

## Introduction

Gold nanoparticles (AuNPs) can be used as diagnostic agents, radiosensitizers and drug delivery vehicles, due to their specific physical and chemical properties, such as strong surface plasmon resonance effect, high stability and low cytotoxicity^1–3^. AuNPs can be readily manufactured in various controllable shapes, sizes and monodispersity. Though non-functionalized AuNPs can show selective cytotoxicity to certain cell lines, especially cancer cells^4^, AuNPs are generally considered nontoxic to normal cells^5,6^. Cytotoxicity of AuNPs is size dependent, small AuNPs elicit higher cytotoxicity than larger AuNP. AuNPs of ~20 nm diameter elicit relatively low cytotoxicity in both normal and cancer cells^7^ and are optimal for traversing the blood brain barrier to enter the brain^8^. The surface chemistry of AuNPs enables bio-conjugation and bio-modification, for example, conjugation of antibodies to assist in targeting or conjugation of chemotherapeutic or detection agents^1,3^. These properties underpin the emergence of gold nanoparticles as promising therapeutic and diagnostic administration systems to treat neoplasms.

Plasma, a form of ionized gas, is one of the four fundamental states of matter and by far the most common form of matter in the universe. Initially, biomedical applications of plasma concentrated on heat and high temperature, i.e. thermal plasmas, for tissue removal, sterilization, and cauterization^9^. Technological advances have allowed researchers to generate plasmas at ambient temperatures and at approximately 1.0 atmospheric pressure, allowing safer application to biological samples and tissues without risking thermal injury. These are known as non-thermal atmospheric plasma (NTAP) or Cold Atmospheric Plasma (CAP). CAP has been investigated as a promising technique for various biomedical applications including tumour therapies, sterilization, wound healing and local viral and microbial infection control^10–13^. CAP generates a unique physical and chemical environment for exposure of biological tissues, eliciting effects such as activation of short and long lived reactive nitrogen species (RNS, e.g. N_2_^+^, NO_3_ and NO, etc.) and reactive oxygen species (ROS, e.g. OH•, O and O_2_•, etc.), photons as well as generation of heat, pressure gradients, charged particles, and electrostatic and electromagnetic fields^14,15^, many of which are known to induce effective death pathways in cancer cells^13^.

Synergistic anti-cancer effects between AuNP and CAP have emerged as a promising potential approach in cancer therapy studies. Kim *et al*. first reported that cytotoxicity of CAP to melanoma cells was significantly increased (near five-fold) by combining with antibody-conjugated AuNP^16^. Zhu *et al*. showed that CAP coupled with drug loaded core-shell AuNP led to a significant enhancement in growth inhibition of breast cancer cells compared with control groups^17^. Other studies have also suggested that AuNP have synergistic cytotoxicity when combined with CAP in cancer treatment^18,19^. However, the effects of CAP and AuNP can vary due to the wide range of different AuNP, CAP-generating devices and cell lines.

**Figure 1:**
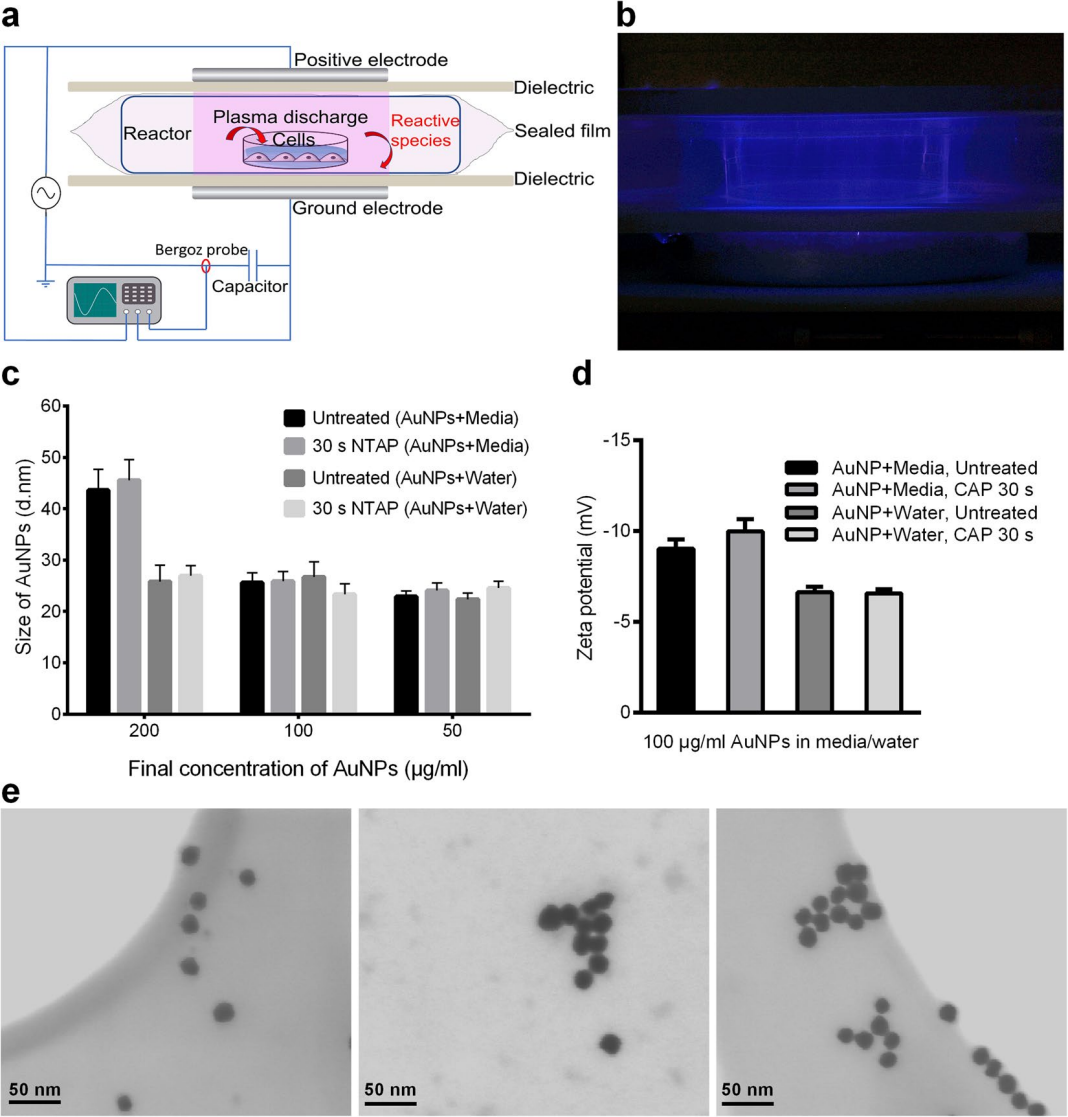
CAP demonstrates no significant effect on AuNPs (≤100 μg/ml) within culture medium or water. (**a**) Schematic of the DIT120 Plasma Device used in this study. (**b**) A photograph showing plasma generation (blue) and treatment on cell samples. (**c**) 20 nm AuNPs were diluted to different concentrations with cell culture medium or water. The mixed solutions were treated with CAP (75 kV, 30 s), incubated for 24 h, then measured the sizes by Zetasizer. (**d**) AuNPs were diluted in cell culture medium or water to 100 μg/ml and treated with CAP at 75 kV for 0, 30 s. Zeta potential was then measured by Zetasizer. (**e**) STEM of AuNP, from left to right: mixed with water; mixed with culture medium; mixed with culture medium then exposed to CAP for 75 kV, 30 s, AuNP concentrations are 100 μg/ml. For Fig. 1c and d, Zetasizer measurements were repeated in six replicates. Statistical analysis was carried out using one-way ANOVA with Bonferroni’s multiple comparisons post-test.

The current research uses an experimental dielectric barrier discharge (DBD) plasma device, DIT 120, with a maximum voltage output of 120 kV at 50 Hz generated between two 15 cm diameter aluminium disk electrodes (Fig. 1a,b)^20,21^. We have previously characterised biological activities in cancer cells that are dependent and independent of reactive species generation using this system^22,23^. We wished to explore the mechanism of CAP and AuNP induced cytotoxicity in cancer cells using our system. Using various analytical chemistry, biochemistry and microscopy to characterise the effects of CAP on AuNP, we provide evidence that direct exposure to CAP induces increased AuNP endocytosis and trafficking to lysosomes, which could be the mechanism of the synergistic cytotoxic effects observed in GBM U373MG cells.

## Results

### The effects of CAP on the physical properties of AuNP

CAP has been used to fabricate metal nanoparticles (e.g. gold and silver) without adding reducing agents^24^. Moreover, AuNPs are known to agglomerate upon stresses such as repeated centrifugation, dilution, dialysis, and upon contact with biological media^25^. We first explored whether exposure of 20 nm AuNP to our experimental plasma device DIT 120 altered their physical parameters (size, shape, surface (zeta) potential, or extent of agglomeration) in water and biological cell culture medium. A concentration range of AuNP (50–200 μg/ml) in water or culture medium was treated with CAP (75 kV, 30 s), or untreated and incubated for 24 hours prior to analysis. As evident in Fig. 1c, agglomeration was evident at higher concentrations (>100 μg/ml) in culture medium and no agglomeration of AuNP was evident even at 200 μg/ml in water. CAP did not induce any significant change in particle size and there is no evidence of agglomeration either in water or in culture medium (Fig. 1c, P = 0.1160 in media group, > 0.9999 in water group). We also confirmed that dilution of AuNPs in culture medium to 100 μg/ml and exposure to CAP (75 kV, 30 s) has no significant change in relation to other physical properties such as zeta potential (Fig. 1d, P > 0.05) or optical absorption (see Fig. S1 in the *Supplementary Information*). Moreover, no discernible change in physical parameters was evident using Scanning Transmission Electron Microscopy, nanoparticles remaining spherical with an average diameter of 20 nm and displaying similar degrees of dispersion across the surface of the gird (Fig. 1e). As AuNP were not observed to agglomerate in culture medium for concentrations AuNP ≤ 100 μg/ml and no changes in physical parameters were detected in response to CAP, culture medium containing up to 100 μg/ml AuNPs were used in subsequent experiments exploring uptake kinetics of AuNP.

### Synergistic cytotoxicity when CAP and AuNP are combined

**Figure 2:**
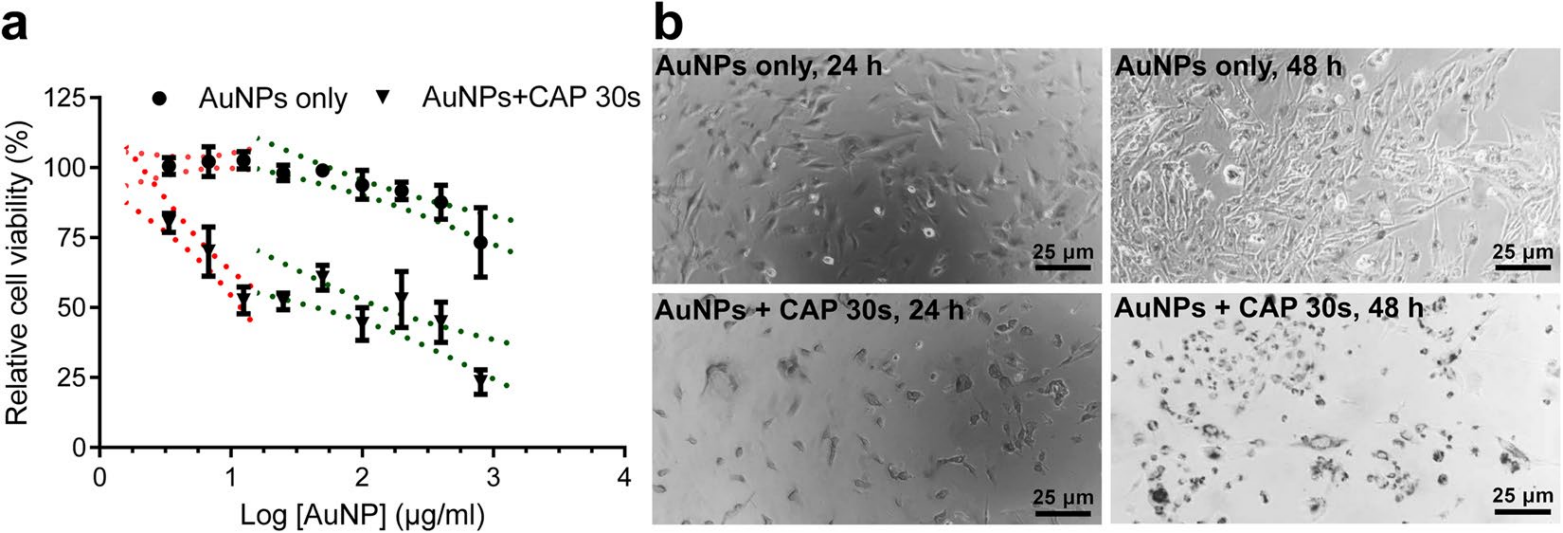
Cytotoxic synergy observed between CAP and AuNP. (**a**) U373MG cells were treated with different concentrations of AuNPs (0–800 μg/ml). After incubation for 48 h, cell viability was analysed using the Alamar Blue assay. The cell viabilities were divided into two phases (red and green) and non-linear regression was carried out. The 95% confidence bands are displayed using dotted lines. Significant differences in the slope (p < 0.05) are evident using non-toxic AuNP concentrations (0–12.5 μg/ml) which was further confirmed using Pearson’s Correlation test (p < 0.05). All experiments were repeated in five replicates. (**b**) After CAP treatment (75 kV, 0, 30 s) U373MG cells were incubated with 100 μg/ml AuNPs for 24 h, 48 h, then observed under optical microscope.

In our previous study, U373MG cells were exposed to CAP for between 3 and 300 s at 75 kV and the IC_50_ value of CAP treatment was determined to be 74.26 s (95% confidence range of 47.24–116.8 s)^22^. The toxicity measured in U373MG cells exposed to 30 s CAP is low (18.52%, SEM = 5.41%) (see Table S1 in the *Supplementary Information*). Figure 2a shows the results of non-linear regression analysis used to calculate IC_50_ values and confidence ranges for cells treated with AuNP alone and AuNP combined with CAP (75 kV, 30 s) (see Table S1 in the *Supplementary Information*). The IC_50_ value for AuNP alone was 2125 μg/ml (95% confidence range: 1294–3491 μg/ml) (Inverse IC_50_ = ~0.471 μl/μg), which is in agreement with other reports that ~20 nm spherical gold nanoparticles have very low cytotoxicity to healthy or cancerous cells^6,7^. The IC_50_ value for AuNP combined with CAP was 81.71 μg/ml (95% confidence range: 53.83–124.16 μg/ml) (Inverse IC_50_ = ~12.238 μl/μg), ~26 times more toxic by comparing their inverse IC_50_. The dose response curve was divided into two phases of toxicity (Fig. 2a) to demonstrate synergy between CAP and AuNP. In the first phase, using low concentrations of AuNP (0–12.5 μg/ml), a sharp and significant drop in viability was observed only when cells were incubated with both CAP and increasing AuNP concentrations. The 95% confidence intervals of the slope of CAP and AuNP treated cells during first phase (−63.57 to −33.43) was significantly different to AuNP only treated cells (−5.573 to 12.71). We believe this provides evidence that CAP and AuNP have a synergistic cytotoxicity to U373MG cells and the uptake of AuNP could reach to a threshold with increase concentration of AuNP.

Morphological assessment using bright-field microscopy confirmed the accumulation of AuNP in cells, but this was significantly greater when cells were pretreated with CAP (Fig. 2b). We therefore hypothesised that CAP can accelerate the uptake and accumulation of AuNPs into U373MG cells, thereby causing higher cytotoxicity. To explore the mechanism of synergistic cytotoxicity of CAP and AuNPs, we used 100 μg/ml AuNPs which is close to the IC_50_ value, at which no discernible physical changes to AuNP was observed.

### Role of active (ATP-dependent) and passive (ATP-independent) transport mechanisms

**Figure 3:**
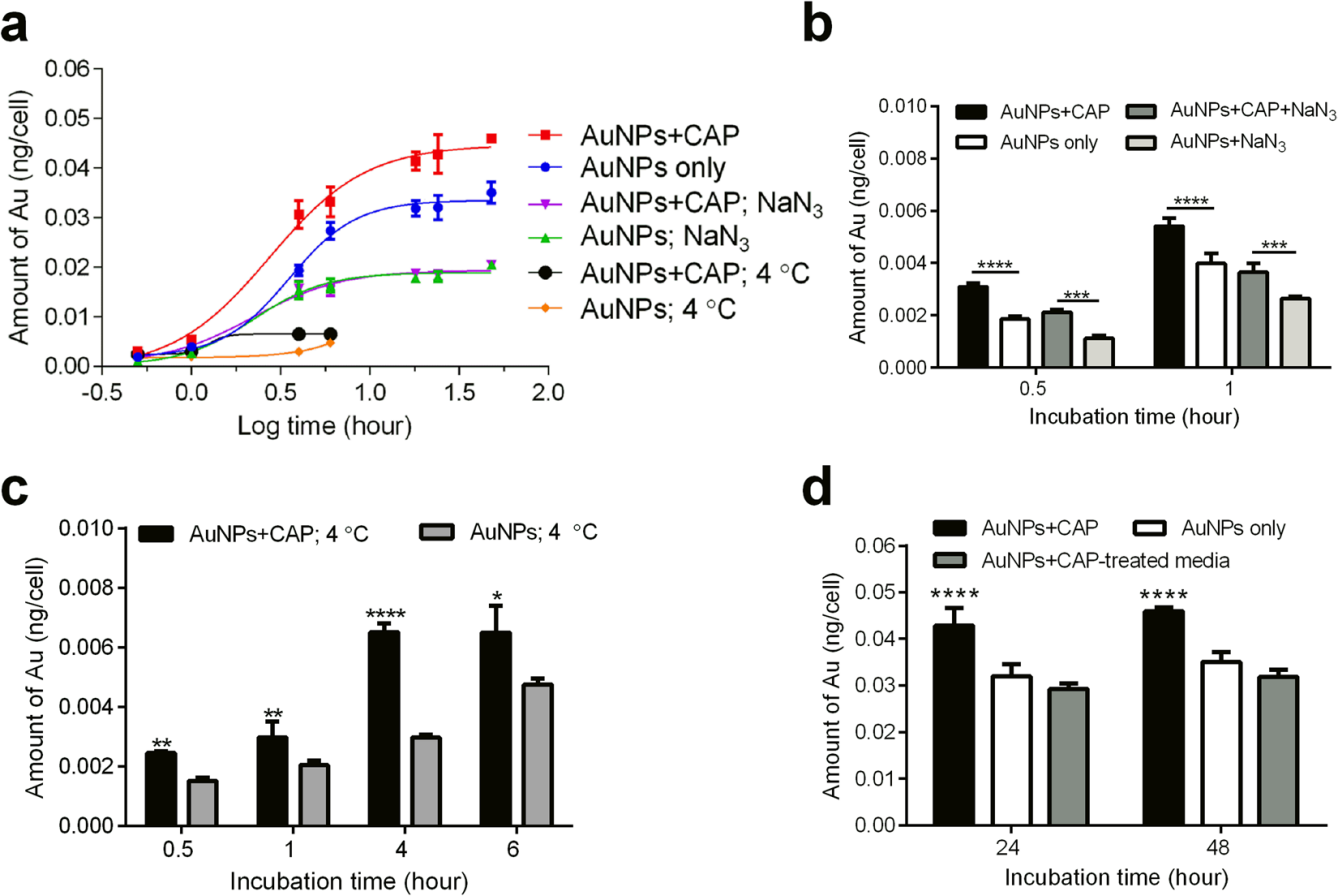
AAS analysis demonstrate the accelerated uptake of AuNPs into cells. (**a**) After CAP treatment (75 kV, 0, 30 s), U373MG cells were treated with 100 μg/ml AuNPs together with 1% NaN_3_ or without to inhibit endocytosis, then incubated at 37 °C or 4 °C as indicated before calculating the average amount of Au per cell using AAS. The uptake curves were assessed by non-linear regression analyses. All experiments were repeated in six replicates. (**b**) The short-time effects of CAP with or without 1% NaN_3_ were compared_._ (**c**) The uptake of AuNPs incubated at 4 °C for 0.5, 1, 4, 6 h. (**d**) The role of long-lived ROS generated by CAP on endocytosis in U373MG cells was determined by using Plasma activated media (PAM; 75 kV, 30 s, stored overnight) in place of CAP. For Fig. 3b–d, all experiments were repeated in six replicates. The statistical significances were assessed by two-way ANOVA with Tukey’s multiple comparison post-test (*P < 0.05, **P < 0.01, ***P < 0.001, ****p < 0.0001).

To confirm the hypothesis that CAP affects enhanced AuNP uptake, and to investigate the mechanisms, Atomic Absorption Spectroscopy (AAS) was used to quantify the total amount of gold in cells. Intracellular AuNP accumulated over time and followed a similar overall non-linear relationship for cells with or without exposure to CAP. We observed that CAP significantly increased both the initial rate of uptake and total amount of AuNP accumulated in cells (Fig. 3a).

To confirm whether active transport or passive diffusion mechanisms were involved, we used the mitochondrial decoupler, NaN_3,_ to identify endocytosis, in which ATP is the rate limiting component. Meanwhile, cold temperature incubation (4 °C) was used to inhibit all energy-dependent uptake in cells. NaN_3_ inhibited AuNP uptake by about 50% when compared with controls (Fig. 3a). Interestingly, NaN_3_ treatment inhibited the uptake of AuNPs in both CAP-treated cells and untreated cells. No significant difference was observed between these two groups between 4 and 48 hours (Fig. 3a), indicating that CAP-stimulated uptake of AuNPs is mainly ATP-dependent. Cells were then incubated at 4 °C for 0.5, 1, 4, 6 h (cell viability was affected at longer time points). As expected, uptake of AuNP was significantly inhibited compared with controls (Fig. 3a). Together, we believe this provides evidence that an active transport mechanism, most likely endocytosis, is the major route of uptake of AuNP in cells, which occurred between 0 and 16 hours after incubation with AuNP, and which was stimulated by CAP exposure. We could not rule out other transport mechanisms playing a minor role. For example, we observed a small fraction (i.e. 10%) of CAP-stimulated AuNP accumulation in cells that was not inhibited in either NaN_3_ or 4 °C-incubated cells. This second, minor CAP-dependent uptake mechanism occurs within 1-hour exposure to NaN_3_ (Fig. 3b) and 4 hours of exposure to 4 °C-incubated cells (Fig. 3c). Plasma-activated culture medium (PAM) containing AuNPs was used to treat cells to determine the role of long-lived reactive species^26^. As seen in Fig. 3d, the uptake of AuNPs in PAM showed no significant difference when compared with the control after 24 and 48 h incubation.

**Figure 4:**
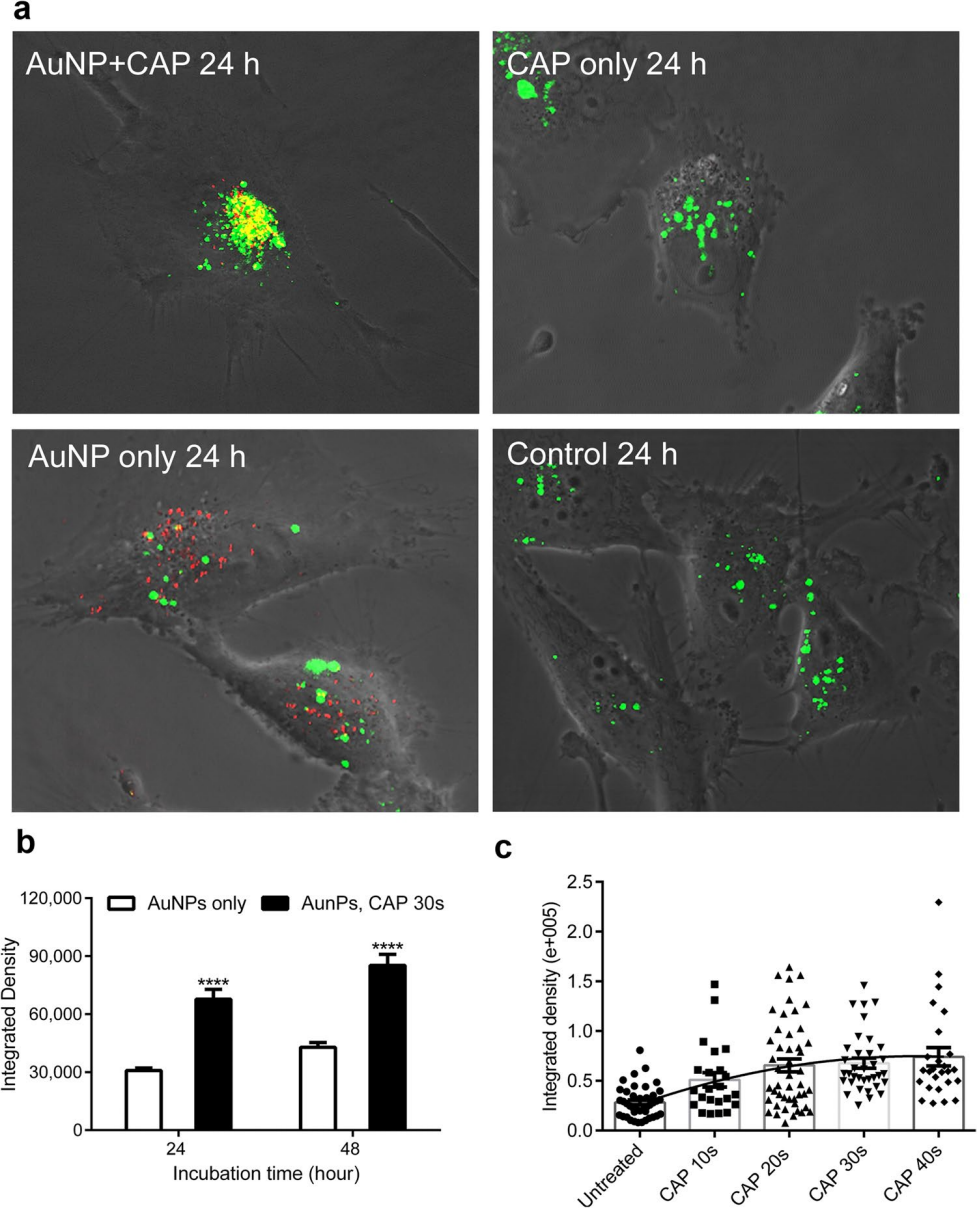
Uptake and subcellular localization of AuNPs observed by confocal microscopy. (**a**) After CAP treatment (75 kV, 0, 30 s), U373MG cells were incubated with 100 μg/ml AuNPs for 24 h. Cell lysosomes were stained using 50 nM LysoTracker™ Green DND-26. The reflection of AuNPs was measured by confocal microscopy. (**b**) After CAP treatment (75 kV, 0, 30 s), U373MG cells were incubated with 100 μg/ml AuNPs for 24 h, 48 h. Cells were then measured by confocal microscopy, the level of reflection of AuNPs was quantified using ImageJ. The statistical significance was assessed by two-way ANOVA with Tukey’s multiple comparison post-test (*P < 0.05, **P < 0.01, ***P < 0.001, ****p < 0.0001), n ≥ 20. (**c**) After exposure to CAP for increasing time (75 kV, 0–40 s), U373MG cells were incubated with 100 μg/ml AuNPs for 24 h. Cells were then measured by confocal microscopy, the level (Integrated Density) of reflection of AuNPs was quantified using the software, ImageJ. Statistical analysis was carried out using Pearson’s correlation, r = 0.9359, *P (two-tailed) = 0.0193, n ≥ 20.

### Subcellular localization of AuNPs endocytosed in response to CAP treatment

Despite the accuracy of using AAS to quantify to total amount of Au in a sample, one limitation with the technique is the difficulty in differentiating between AuNP that are loosely associated with the plasma membrane compared with intracellular AuNP. To verify the uptake of intracellular AuNP, investigate the subcellular location and quantify the rate of uptake, we used confocal microscopy (Fig. 4). Unlabelled, citrate-capped AuNP were visualised using reflection of laser light from the colloid gold (red dots) and lysosomes were counterstained using LysoTracker Green, as shown in Fig. 4a. The absorption spectrum of 20 nm AuNPs confirmed that the absorption peak is around 520 nm, and that there is minimal absorbance above 600 nm (see Fig. S1 in the *Supplementary Information*). The reflection filter setting for AuNPs was set at 649–799 nm and the emission filter for LysoTracker Green was set at 505–530 nm. This allowed us to confirm that the green fluorescence signal is from LysoTracker Green (excited with argon laser) and red reflection is from AuNP (reflected light from HeNe laser). A significant fraction of intracellular colocalization was evident (yellow), indicating that the lysosomal compartment was the major destination of AuNP following CAP-stimulated uptake (Fig. 4a). Interestingly, some AuNPs were not located in lysosomes, suggesting that these AuNPs remained in early endosomes or had entered the cytosol, either by passive diffusion, transport mechanisms through the cell membrane or released from lysosomes subsequent to endocytosis. Significantly higher levels of intracellular AuNP in cells were observed following CAP treatment (Fig. 4b), in agreement with our AAS data. Moreover, varying the dose of CAP confirmed a significant, strong and positive correlation exists between exposure to CAP and subsequent AuNP uptake (Fig. 4c), Pearson’s correlation, r = 0.9359, P = 0.0193.

**Figure 5:**
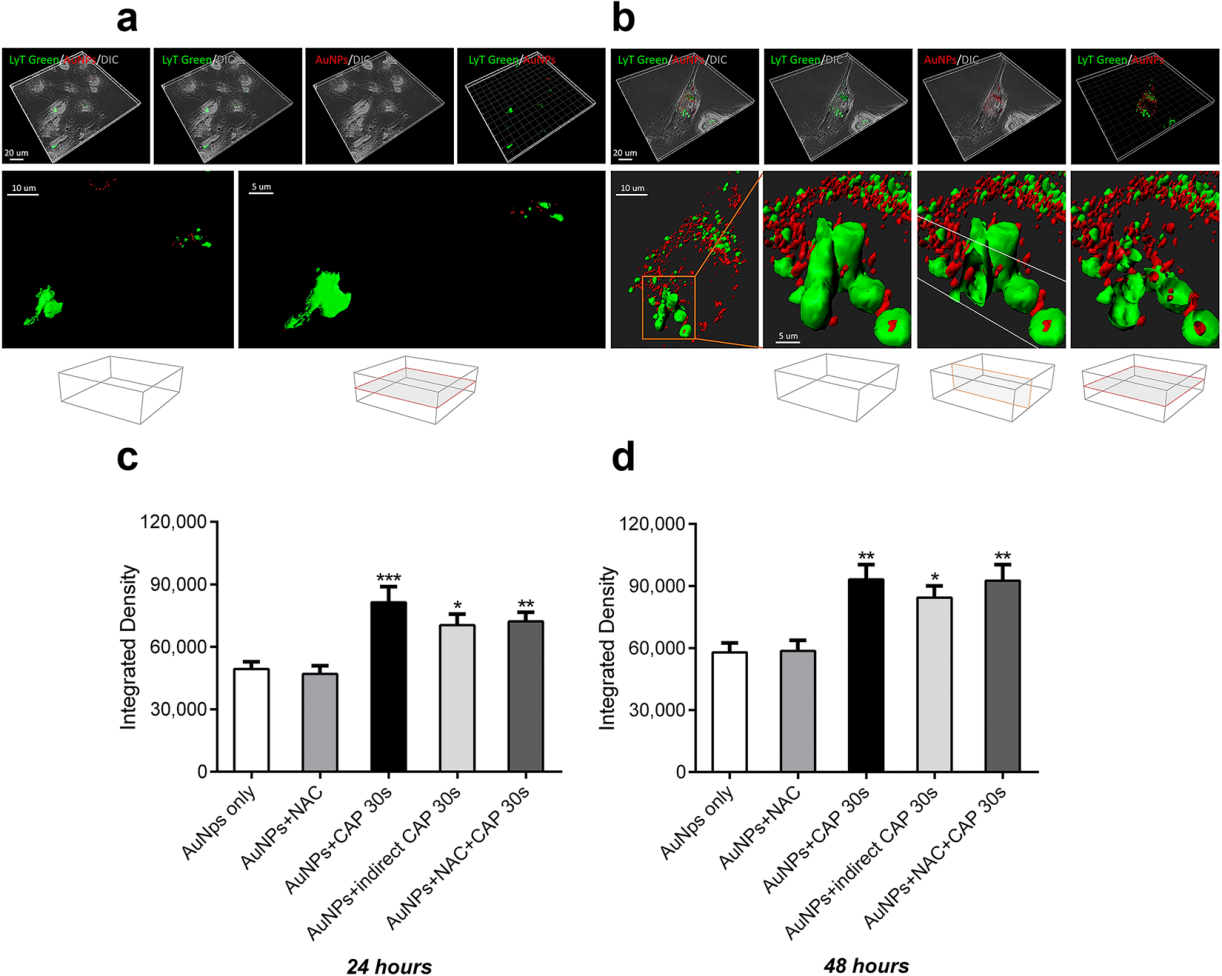
AuNPs are incorporated within lysosomes in glioma cells. (**a**) 3D reconstruction of the untreated glioma cells visualized with dichroic channel (grey), incorporating AuNPs (red) within lysosomes (LysoTracker green). U373MG cells were incubated with 100 μg/ml AuNPs for 24 h, after CAP treatment (75 kV, 0, 30 s). Low incorporation of AuNPs inside the cells is demonstrated. High magnifications in bottom panel show lysosomes evidenced with green isosurface. Vertical and horizontal clipping planes reveal no red material inside lysosomes. Boxes at the bottom indicate the orientation of the clipping planes (**b**) Analogous 3D reconstruction of CAP-treated cells, demonstrated a significant increase of AuNPs in the entire cells and in part located inside lysosomes. High magnification in the bottom panel show lysosomes evidenced with green isosurface. Vertical and horizontal clipping planes reveal red AuNPs nanoparticles inside lysosomes. High magnification at the bottom panel shows lysosomes rendered with green isosurface. (**c,d**) The role of short and long-lived reactive species generated by CAP on endocytosis in U373MG cells was determined by pretreating cells with 4 mM anti-oxidant N-Acetyl Cysteine (NAC) for 1 h before CAP treatment (75 kV, 30 s) or by indirect CAP treatment (see detail in Method). The level of reflection of AuNPs was quantified using the ImageJ after incubated for 24 (**c**) or 48 hours (**d**). The statistical significance was assessed by two-way ANOVA with Tukey’s multiple comparison post-test (*P < 0.05, **P < 0.01, ***P < 0.001), n ≥ 20.

Z-stacked images were obtained to ensure that only intracellular AuNP were quantified. Stacked, deconvoluted confocal images were next reconstructed using rendering software (Imaris 8.0. Bitplane) to generate 3 dimensional reconstructions of individual cells. The presence of AuNP (red) inside lysosomes (green) was confirmed by rotating and sectioning the cells around the three spatial axes and projecting sections from the x, y and z planes of the reconstructions (Fig. 5a,b). Taken together, our data confirm that CAP induces synergistic cytotoxicity while-stimulating the uptake of citrate capped 20 nm AuNP through a predominantly endocytic mechanism, leading to trafficking of the AuNP into acidic (lysosomal) compartments of U373MG cells.

### Role of physical and chemical effects of CAP on AuNP uptake

Our data indicated that long-lived reactive species present in plasma activated media (PAM) did not play a major role in enhanced uptake of AuNP (Fig. 3d). We wished to further investigate effects of CAP on AuNP endocytosis. Due to the set-up of the DBD plasma device, samples can either be placed directly in the plasma discharging area (direct exposure) or outside the plasma-discharging area (indirect exposure). Plasma sources for direct or indirect plasma treatment were previously distinguished by Fridman *et al*.^9^ and more recently by Von Woedtke *et al*.^27^. In indirect plasma treatment, the electrodes are part of the plasma-generating device, only and there is no significant electrical contact to the targeted structures, whereas in direct plasma treatment, the biological samples serve as one of the two electrodes. In both cases, samples are contained within a sealed reactor. Direct exposure results in direct interaction with UV, electric fields, electron beams, charged particles, whereas indirect exposure does not result in any interaction with these physical species. However, the sealed reactor system still allows the exposure of reactive species to cells. It has been demonstrated that ozone concentration and reactive oxygen species generated are not significantly different during direct or indirect CAP exposure^21,28^. We investigated the difference between direct and indirect CAP treatment on AuNP uptake to differentiate between direct physical effects and chemical (oxidation) effects. Our results demonstrated that AuNP uptake was significantly higher when cells were treated with indirect CAP exposure compared with untreated cells, suggesting that chemical effects were important in promoting endocytosis (Fig. 5c,d). The enhanced AuNP uptake was consistently slightly lower compared with direct CAP exposure although we were not able to determine a significant difference, suggesting that any physical effects only play a minor role in the increased uptake of AuNP. We used N-Acetyl Cysteine (NAC), a synthetic precursor of intracellular cysteine and glutathione (GSH) that replenishes intracellular GSH and scavenges reactive species as a redox buffer^29^ to remove long-lived ROS *in situ* during CAP treatment. This was ineffective in significantly reducing AuNP uptake enhanced by CAP (Fig. 5c,d), suggesting that either the oxidising environment generated by CAP overwhelms GSH and other intracellular anti-oxidant defences, or that NAC-insensitive chemicals produced by both direct and indirect contained CAP fields are the primary cause of enhanced AuNP uptake.

## Discussion

AuNPs have been developed as promising theranostic agents for brain cancer therapy in diverse applications, such as *in vivo* tumour imaging, inducing radiosensitization and targeted delivery of chemotherapeutics across blood-brain barrier (BBB) to brain cancer cells^1,30^. As an emerging platform for drug delivery, the toxic effect of AuNP to normal cells can be minimized or eliminated by altering the size^7^. However, the proportion of AuNPs that penetrate the BBB is usually no more than 1%^31^. To date, efforts to enhance AuNP crossing of the BBB have been mainly focused on targeting AuNP to the surface receptors of endothelial cells^32^ and the limited success means that there is still need for further studying the mechanisms of crossing BBB and blood-tumour barriers, etc. The permeability, targeting capacity and uptake of AuNP into targeted cancer cells are the key to success clinical application of AuNP. CAP treatment has been extensively investigated in cancer therapy, due to its promising selective capacity of killing a wide range of cancer cells^13^, such as carcinomas, glioblastomas, melanomas and hematopoietic malignancies^16,33,34^. In recent years, CAP has been successfully and safely used in a prospective clinical trial for head and neck cancer treatment and chronic wound healing, demonstrating significant benefits and no side-effects. Meanwhile, the synergistic anti-cancer effects between AuNP and CAP were reported in several previous studies^16–19,35^.

Although cancer cells are generally reported to be favourably sensitive to CAP induced cytotoxicity when compared with normal cells or tissues^14^, U373MG GBM cells have significantly higher resistance to CAP treatment compared to other cancer cell lines^22^. GBM cells also show high aggressiveness and resistance to radiation therapy and most chemotherapies^36^. There is evidence in the literature that AuNPs have synergistic cytotoxicity combined with CAP, although the mechanisms remain to be elucidated. Therefore, we chose to study the mechanism of CAP, combined with AuNP, as a possible future chemotherapy delivery vehicle. In this study, we confirmed that combining CAP with AuNP increased around 25-fold of U373MG cell death compared to AuNP only. We also demonstrated that CAP treatments accelerate the endocytosis of U373MG cells by temporarily increase membrane permeabilisation or turnover, thereby increasing uptake and cytotoxicity of AuNP. These effects will be useful to induce higher selective cytotoxicity against cancer cells while increasing drug delivery efficiency and/or imaging diagnostics for cancer therapy.

In this study, we used 20 nm citrate capped AuNPs, which not only have optimal BBB permeability and low toxicity, but also can rapidly enter cells, mostly by receptor-mediated endocytosis^7,8,37^. Therefore, 20 nm AuNPs can be the optimal candidate for drug delivery across BBB. We were able to confirm that the observation of the uptake of 20 nm citrate capped AuNPs are consistent with endocytosis and a large proportion of them were trapped in lysosomes in both CAP-treated and untreated U373MG cells (Figs 3, 4 and 5). We confirmed that CAP did not discernibly alter the physical properties of AuNP, although we did see evidence that CAP could stimulate low levels of non-active uptake through an ATP-independent mechanism, which may indicate some level of surface modification or other physical change to AuNP and/or cell membranes. Although we were unable to detect physical changes to AuNP exposed to CAP, significant alterations in cell membranes after exposure to CAP were previously observed by others^38^. Our observation that synergistic cytotoxicity occurs in parallel with enhanced uptake suggests the two processes are linked in U373MG cells. Although, there have been no studies reported to date on the relative sensitivity of normal and GBM cells to CAP-induced endocytosis, it has been demonstrated that the significant alterations in cell membrane of GBM cells induced by CAP treatment is maintained, whereas the membrane alteration in normal human astrocytes E6/E7 is weaker and reversible^38^. More detailed mechanisms regulating the uptake of nanoparticles in normal and GBM cells will be investigated in a follow-on study. A controllable, directable CAP treatment, which is directly applied to diseased tissue, also is expected to be included to present the *in vivo* experiment in the follow-on study. At this stage, we provided a likely hypothesis as follows.

It is possible that enhanced uptake of AuNP may underpin the synergistic cytotoxicity observed, in combination with other effects such as the higher load of reactive species carried by CAP-treated AuNP. For example, it has been reported that AuNP are capable of trapping reactive species and extending their half-life, and thus helping the delivery of CAP generated reactive species into cancer cells^15^. It is also likely that the enhanced accumulation of AuNP is caused by one of three effects attributed to CAP, i.e. reactive species, direct physical effects and cellular mechanism (mainly endocytosis).

Considering the potential role of reactive species, hydrogen peroxide (H_2_O_2_) was found to be capable of facilitating the endocytosis of polyethylenimine/oligonucleotide (PEI/ON) complexes, involving a cytoplasmic [Ca^2+^]-independent activation of calcium/calmodulin-dependent protein kinase II (CaMKII), which stimulates cytoskeleton contractions and transportation^39^. It was also found that the ROS generated in mitochondria mediate the hypoxia-induced endocytosis in alveolar epithelial cells^40^. It is considered that CAP is capable of influencing cell behaviour by generating intracellular ROS and RNS^22^. These studies imply that the CAP-generated intracellular ROS could have an impact on the enhanced endocytosis. Meanwhile, reactive species are capable of inducing membrane damage and increasing the permeability of cell membrane^41^. The reactive species generated in the cytoplasm or in the media can induce lipid peroxidation in membranes by oxidizing the polyunsaturated fatty acids. Reactive species are also able to attack membrane proteins, causing the membrane damage^41^. It has been found that the membrane lesions are capable of activating rapid endocytosis to remove damage parts and preserve and repair the integrity of cell membrane^42^. Therefore, it is possible that the chemical and physical membrane damage caused by CAP could activate the membrane repair response, thus accelerating the endocytosis of AuNPs. Although we found that long-lived reactive species have little effect on the uptake of AuNP (Fig. 3d), the short-lived, highly reactive species may play a role in the stimulated uptake, which will need to be further elucidated.

Considering the direct physical effects, which are mainly electrical factors in DBD CAP treatment, Jinno, M *et al*. has described the roles of chemical (reactive species) and electrical effects of CAP in the CAP gene transfection in L-929, mouse fibroblast cells^43^. It was found that reactive species alone do not work, but need to be combined with electrical effects. Electroporation effects, which can last for a few minutes, were also found to play a role in gene transfection^43^. Computational modelling was used to determine that large pore formation in spherical cell membranes (i.e. 15–25 nm) can be induced in strong electric fields *in silico*^44^, a notion which has since been confirmed for 20 nm AuNP *in vitro*^45^. Meanwhile, it was observed that the treatment of CAP in U87 cells results in an uneven membrane and development of membrane pores^46^. Nina Recek *et al*. have studied the differential effects of CAP on cell membranes of normal human astrocytes (E6/E7) and U87 cells by atomic force microscopy. CAP treatment causes a temporary disappearance of microvilli in E6/E7, and unrecoverable partial cell membranes and cell components damage of U87 cells. The temporary pores created in membranes and membrane damage could be the ATP-independent mechanism that stimulates low levels of non-active uptake of AuNP during the first few hours after treatment with NaN_3_ or under low-temperature incubation (Fig. 3b,c). Moreover, it also known that CAP is capable of temporarily changing the polarity/potential of the membrane, which also could play a role in stimulating endocytosis^47^. As seen in Fig. 5c,d, the indirect treatment, which removes the direct physical effects caused by CAP, showed only slightly decreased accumulation of AuNP and no significant difference evident compared with direct treatment. It confirmed that the temporary direct physical influence caused by DBD CAP treatment plays only a minor role in the increased uptake of AuNP.

It is considered that the endocyosis of cells can be stimulated by CAP via reactive species and other mechanisms such as membrane damage, as indicated above^39,42,43^. In gene transfection, the clathrin-dependent endocytosis stimulated by CAP was found to be dominant in the uptake, while synergistically combining with electrical effects^43^. We observed two phases to the enhanced uptake of AuNP. Over the first one hour, we observed both increased endocytosis and possibly also increased passive diffusion through damaged or otherwise permeable membranes (Fig. 3b,c). We then observed a second phase, between 4 and 18 hours, where AuNP uptake was further accelerated by CAP primarily though endocytosis with no evidence for further passive mechanisms before reaching a maximum accumulated threshold (Fig. 3a).

Taken together, we report that the synergistic cytotoxicity of AuNP and CAP is a result of enhanced endocytosis and trafficking to the lysosomal compartment as well as temporarily increased membrane permeability, due to CAP treatment. This contributes understanding to the mechanisms of synergistic cytotoxic effects between CAP and nanotechnologies and identifies strategies that may be employed for the release of drugs when used in a drug-delivery capacity.

## Methods

### Cell Culture

The human brain glioblastoma cancer cell line (U373MG-CD14) cells were obtained from Dr Michael Carty (Trinity College Dublin). Cells were cultured in Dulbecco’s Modified Eagle’s Medium-high glucose (Sigma-Aldrich) supplemented with 10% foetal bovine serum (Sigma-Aldrich) and 1% penicillin and streptomycin mixture (Thermo Fisher Scientific) in TC flask T25, standard for adherent cells (Sarstedt). The cultures were maintained under a condition of 5% (v/v) CO2 and 37 °C in a humidified incubator. Culture medium was changed every 2 days until reaching around 80% confluency. Cells were then brought into suspension using 0.25% trypsin solution (Thermo Fisher Scientific) and subcultured in new flasks.

### CAP Configuration and Treatment

The CAP-DBD device used (Fig. 1a,b) is an experimental atmospheric low temperature plasma generator^22^. It consists of two aluminium disc-electrodes (diameter 15 cm) with a polypropylene sheet in the middle, which is used as dielectric barrier and holder for the reactor and cell samples. The thickness of the dielectric barrier is 1.2 mm, and the distance between the two electrodes was 26.6 mm. Samples were placed in a sealed reactor and treated inside or outside the plasma discharging area. The CAP was generated between two disc-electrodes when the high voltage was applied. Voltage was monitored using an InfiniVision 2000 × -Series Oscillo-scope (Agilent Technologies Inc., Santa Clara, CA, USA). TC dish 35 standard (35 × 10 mm, Sarstedt) was used as the cells container for CAP treatment. U373MG cells were seeded into the dishes at a density of 1 × 10^5^ cells/ml and incubated overnight to allow a proper adherence (70–80% confluency). For direct treatment, culture medium was removed from the dishes before the CAP treatment, then the cell culture dishes were put between the two electrodes and treated at 75 kV for 0–40 s. The fresh culture medium containing 100 μg/ml AuNPs or inhibitors was added after exposing to CAP. For CAP-activated culture medium, the fresh culture medium were contained in TC dish 35, standard and treated with CAP at 75 kV for 30 s and stored overnight to remove short-live reactive species. AuNPs were added into CAP-activated media to 100 μg/ml. The CAP-untreated cells were then incubated with CAP-activated media containing AuNPs for 24 and 48 h. The indirect CAP treatment was used to determine the role of direct physical effects. To indirectly treat cells with CAP, the sample was treated in the reactor without the dish lid on it, and culture medium was removed before the treatment. The cell culture dish was placed on the corner of the reactor, outside the plasma discharging area. During the treatment (75 kV, 30 s), the reactor was sealed in a high barrier polypropylene bag (B2630; Cryovac Sealed Air Ltd, Dunkan, SC, USA) to keep the CAP inside the reactor, and to make sure the cells were only affected by the short-lived and long-lived reactive species generated in air^28^. Fresh culture medium containing 100 μg/ml AuNPs was then added after indirect CAP treatment.

### Gold Nanoparticles Synthesis and Characterization

20 nm AuNPs used in this study are synthesised by trisodium citrate (Na_3_C_6_H_5_O) reduction of auric acid (HAuCl_4_). The typical method of AuNPs synthesis as follows. HAuCl4 was dissolved in water at a concentration of 0.25 mM in a clean glass flask and heated with magnetic stirring and brought to boiling. The corresponding amount of 5% (w/v) sodium citrate solution was quickly added to final 3.5:1 molar ratio of citrate to Au3 + while being keep heating and stirring. The reaction was completed until the colour of solution was changed from dark purple to wine red and keep stable. The gold colloid was then centrifuged at 10000 g for 10 min to concentrate the AuNPs stock solution to 2500 μg/ml. The size, absorbance curve and zeta potential of AuNPs was then determined by UV-Vis spectrometer (Shimadzu, UV-1800), Zetasizer (Malvern, Nano ZS) and scanning transmission electron microscopy (STEM). The AuNPs stock solution was diluted in water or culture medium to corresponding concentration as indicated in the relevant figures.

### Cell Viability Assays

Cell viability was analysed using the Alamar blue assay (Thermo Fisher Scientific), which used a redox indicator that can generate fluorescent signal by the metabolic reduction. U373MG cells were plated into 96-well plates (Sarstedt) at a density of 1 × 10^4^ cells/well (100 μl culture medium per well), and were incubated overnight to allow a proper adherence. The cells were then directly treated with CAP at 75 kV for 0–30 s at 70–80% confluency, and culture medium containing 0–100 μg/ml AuNPs was added post CAP treatment. Forty-eight hours late, the cells were rinsed once with phosphate buffered saline (Sigma-Aldrich), incubated for 3 h at 37 °C with a 10% Alamar blue/90% culture medium solution. The fluorescence was then measured (excitation, 530 nm; emission, 595 nm) by a Victor 3 V 1420 microplate reader (Perkin Elmer).

### Atomic Absorption Spectroscopy

U373MG cells were seeded into 60 mm dishes (Sarstedt) at a density of 1 × 10^5^ cells/ml and incubated for 2 days to achieving 90–100% confluency. The culture medium was removed and cells were directly exposed to CAP at 75 kV for corresponding time. The fresh culture medium containing 100 μg/ml AuNPs or 0.1% (w/v) NaN_3_ was then replaced and incubated for 0.5–48 h at 37 °C or 4 °C as indicated. After treatment, cells were washed thrice with phosphate buffered saline to remove the AuNPs outside cells. Cells were then dissociated and collected from the culture dish using prewarmed (37 °C) 0.25% trypsin solution. The cell suspension was counted using hemocytometer, then transferred to 15 ml tube (Sarstedt) to measure the gold atomic absorbance in AAS. To verify the Au amount of the samples, five-point standard curve were first established using 1–5 ppm standard gold colloid. The concentrations of AuNPs in samples were then calibrated by the standard curve as described elsewhere^48^.

### Inhibitor Studies

To determine the uptake of AuNPs, NaN_3_ was used as metabolic inhibitor to inhibit the energy-dependent endocytosis. 20% (w/v) NaN_3_ stock solution was prepared in phosphate buffered saline. The cells were pre-incubated with 0.1% (w/v) NaN_3_ in prewarmed (37 °C) culture medium for 1 h. The culture medium was then removed during CAP treatment, then fresh media containing 0.1% (w/v) NaN_3_ and 100 μg/ml AuNPs was added into the cell culture and incubated at 37 °C as indicated. 4 °C incubation was used to inhibit endocytosis as a standard experiment. Cells were incubated at 4 °C for 1 h before the CAP treatment, pre-chilled culture medium containing 100 μg/ml AuNPs was then replaced post CAP treatment and incubated at 4 °C for 0.5–6 h as indicated. N-Acetyl Cysteine (NAC) was used as antioxidant to remove long-lived reactive species. 4 M NAC stock solution was prepared in water. The cells were pre-incubated with 4 mM NAC in culture medium for 1 h at 37 °C. The culture medium was then removed during CAP treatment. Afterward, cells were incubated in fresh media with 4 mM NAC and 100 μg/ml AuNPs for 24 and 48 h.

### Fluorescent Dyes and Cell Imaging

Lysosomes was demonstrated using the LysoTracker™ Green DND-26 (Thermo Fisher Scientific). Cells were seeded in 35 mm glass-bottom dishes (Greiner Bio-One) at a density of 1 × 10^5^ cells/ml overnight and treated with CAP for 0–40 s at 70–80% confluency, the fresh media containing 0–100 μg/ml AuNPs was then replaced and incubated for 24 h or 48 h at 37 °C as indicated. After treatment, the cells were rinsed thrice with phosphate buffered saline and incubated with prewarmed (37 °C) LysoTracker-containing (50 nM) media for 5 min at 37 °C. Cells were then washed once with phosphate buffered saline and loaded with fresh phosphate buffered saline and observed using Zeiss LSM 510 confocal laser scanning microscope fitted with the corresponding filter settings as follows. LysoTracker™ Green DND-26, excitation wavelength: 488 nm, emission filter: 505–530 nm; AuNPs, excitation wavelength: 633 nm, reflection filter: 649–799 nm. Plan-Apochromat 63 × /1.4 Oil Ph3 was used as objective for all samples. To determine the level of AuNPs reflection, around 50 cells were randomly imaged for each treatment condition and the levels of reflection was analysed using the ImageJ and compared with other groups.

### Statistical Analysis

All data points were from at least triplicate independent samples and are presented and/or pooled as mean ± S.E.M unless indicated otherwise. Curve fitting and statistical analysis were carried out using Prism 6 (GraphPad Software). The Alpha for all tests is 0.05 and two-tailed P values were used. One-way ANOVA and two-way ANOVA was used to verify the significance between data points as indicated in figures (*P < 0.05, **P < 0.01, ***P < 0.001, ****p < 0.0001).

## Electronic supplementary material

Supplementary Information
