# Supplementary material for: Cold Atmospheric Plasma Induces ATP-Dependent Endocytosis of Nanoparticles and Synergistic U373MG Cancer Cell Death
Source: Sci Rep. 2018 Mar 28;8:5298. doi: 10.1038/s41598-018-23262-0 (PMC5871835; doi:10.1038/s41598-018-23262-0)
Supplement: Supplementary file 1 — Supplementary Information [file 41598_2018_23262_MOESM1_ESM.pdf]

TITLE:

Supplementary Information:

Cold Atmospheric Plasma Induces ATP-Dependent Endocytosis of Nanoparticles and Synergistic U373MG Cancer Cell Death

AUTHORS AND AFFILIATIONS:

Zhonglei He[1,2], Kangze Liu[1,2]*, Eline Manaloto[1,2]*, Alan Casey[2], George Paul Cribaro[4], Hugh J. Byrne[2], Furong Tian[1,2,3], Carlos Barcia[4], Gillian E. Conway[1,2], Patrick J Cullen[1,5], and James F. Curtin[1,2,3]

[1]BioPlasma Research Group, School of Food Science and Environmental Health, Dublin Institute of Technology, Dublin, Ireland; [2]Nanolab, FOCAS Research Institute, Dublin Institute of Technology, Dublin, Ireland; and [3]Environmental, Sustainability and Health Research Institutes, Dublin Institute of Technology, Dublin, Ireland; [4]Institut de Neurociències & Department of Biochemistry and Molecular Biology, School of Medicine, Universitat Autònoma de Barcelona, Barcelona, Spain; [5]Department of Chemical and Environmental Engineering, University of Nottingham, UK

*These authors contributed equally to this work.

Corresponding author

Correspondence to James Curtin (james.curtin@dit.ie).

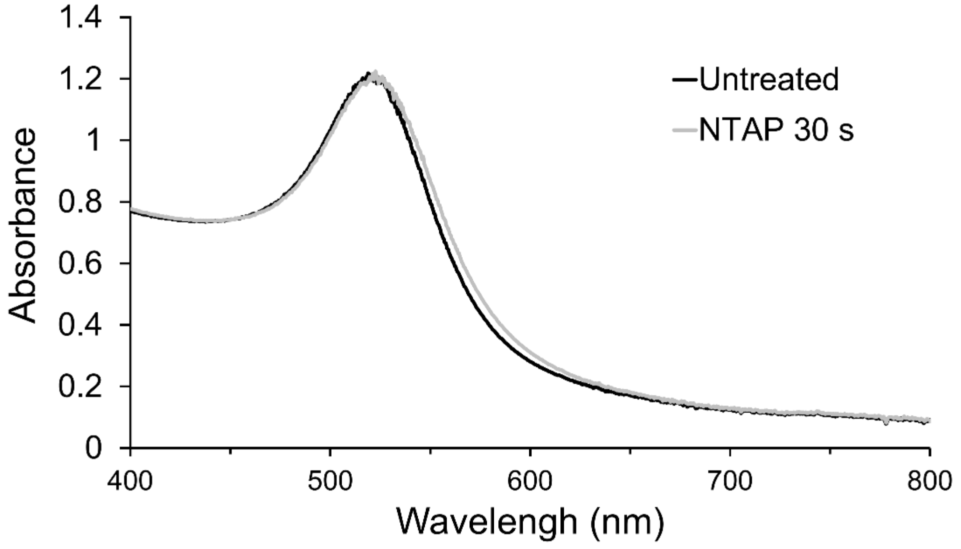

Figure S1. UV-vis absorption spectrum of 100 µg/ml AuNPs dispersed in water. The AuNPs solutions were treated with CAP at 75 kV for 30 s, or untreated.

Raw dataset of the Alamar Blue assay										
AuNP Concentration	Treatment: AuNPs only					Treatment: AuNPs+CAP 30s,75 kV				
800 µg/ml	223	209	289	283	301	113	86	79	81	103
400 µg/ml	292	287	312	321	336	175	122	172	171	183
200 µg/ml	307	325	334	331	320	177	140	219	206	219
100 µg/ml	300	336	337	336	344	164	136	150	184	178
50 µg/ml	335	351	353	352	349	232	210	206	207	238
25 µg/ml	329	346	352	352	346	182	200	198	192	177
12.5 µg/ml	358	356	347	366	374	167	192	210	186	200
6.75 µg/ml	330	356	363	365	379	204	246	277	247	276
3.375 µg/ml	338	348	363	358	360	284	299	269	292	280
0	335	354	352	356	360	242	280	354	292	276
Negative control	13	14	13	14	13					

Table S1. Raw dataset of the Alamar Blue assay. U373MG cells were treated with different concentrations of AuNPs (0-800 µg/ml). After incubated for 48 h, cell viability was analysed using the Alamar Blue assay. The table presented the raw date produced by the Microplate Reader, Synergy HT (BioTek).
